# Flexible Cotton Fabric-Based Ionizing Radiation Dosimeter for 2D Dose Distribution Measurements over a Wide Dose Range at High Dose Rates

**DOI:** 10.3390/ijms25052916

**Published:** 2024-03-02

**Authors:** Marek Kozicki, Elżbieta Sąsiadek-Andrzejczak, Radosław Wach, Piotr Maras

**Affiliations:** 1 Department of Mechanical Engineering, Informatics and Chemistry of Polymer Materials, Faculty of Materials Technologies and Textile Design, Lodz University of Technology, Żeromskiego 116, 90-543 Lodz, Poland; elzbieta.sasiadek@p.lodz.pl; 2 Institute of Applied Radiation Chemistry, Faculty of Chemistry, Lodz University of Technology, Wroblewskiego 15, 93-590 Lodz, Poland; radoslaw.wach@p.lodz.pl; 3 Department of Radiotherapy Planning, Copernicus Hospital, Pabianicka 62, 93-513 Lodz, Poland; piotr.maras@wp.pl

**Keywords:** 2D dosimeter, flexible dosimeter, cotton-based dosimeter, nitrotetrazolium blue chloride, linear accelerator

## Abstract

This work presents an ecological, flexible 2D radiochromic dosimeter for measuring ionizing radiation in the kilogray dose range. Cotton woven fabric made of cellulose was volume-modified with nitrotetrazolium blue chloride as a radiation-sensitive compound. Its features include a color change during exposure from yellowish to purple-brown and flexibility that allows it to adapt to various shapes. It was found that (i) the dose response is up to ~80 kGy, (ii) it is independent of the dose rate for 1.1–73.1 kGy/min, (iii) it can be measured in 2D using a flatbed scanner, (iv) the acquired images can be filtered using a mean filter, which improves its dose resolution, (v) the dose resolution is −0.07 to −0.4 kGy for ~0.6 to ~75.7 kGy for filtered images, and (vi) two linear dose subranges can be distinguished: ~0.6 to ~7.6 kGy and ~9.9 to ~62.0 kGy. The dosimeter combined with flatbed scanner reading and data processing using dedicated software packages constitutes a comprehensive system for measuring dose distributions for objects with complex shapes.

## 1. Introduction

The use of ionizing radiation in research and industry requires careful monitoring and measurement of both radiation dose and dose distribution. In such cases, various types of one-, two-, and three-dimensional radiation dosimeters are used. This work is focused on two-dimensional (2D) dosimeters with unique features: they are made of a natural, common fiber and are flexible. The closest measurement capabilities to the reported systems are 2D film dosimeters.

2D film dosimeters are most often plastic structures with embedded radiation-sensitive compounds. Under the influence of ionizing radiation, compounds change chemically, creating a color whose intensity depends on the absorbed dose. As reported elsewhere, a range of such products with different functions is being investigated [1,2]. Many radiation-sensitive compounds can be used to prepare such dosimeters. For example, a group of tetrazolium salts transforming into colored formazans [3,4,5,6,7] have been intensively studied as components of poly(vinyl alcohol) films in the kilogray dose range, such as 2,3,5-triphenyltetrazolium chloride [8], nitro-blue tetrazolium chloride [9], ethyl violet-bromophenol blue dyed [10], 2,3,5-triphenyltetrazolium chloride, neotetrazolium chloride, 5-(p-nitrophenyl)-2,3-diphenyltetrazolium bromide, 2,5-diphenyl-3-(1-naphthyl)tetrazolium bromide, and 2,2’-di(p-nitrophenyl)-5,5’-diphenyl-3,3’-(3,3-dimethoxy-4,4’-diphenylene)-2H-ditetrazolium chloride (nitrotetrazolium blue chloride, NBT) [11]. Other films were also used to prepare dosimeters, such as polyvinyl butyral with nitro-blue tetrazolium [12] or methyl red [13]. In turn, other researchers focused on investigating the radiotherapy dose range (gray range) film dosimeters (radiochromic and thermoluminescent) [14,15]. Examples of such dosimeters are the silver halide film dosimeter (XV and EDR-2, Kodak, Rocheter, NY, USA) and the EBT radiochromic film dosimeter (GafChromic^®^ EBT, Ashland, Wilmington, DE, USA). Overall, these plastic dosimeters are the subject of numerous studies covering manufacturing, characterization, and clinical applications [16,17,18,19,20,21,22]. The introduction of magnetic resonance imaging combined with linear accelerators (MR-Linac) into radiotherapy [23,24,25] has become an impulse for research related to dose measurements for this type of instrument. In this context, a BCF-60 plastic scintillation detector (PSD) coupled to a PMMA fiber optic cable was proposed to provide real-time dose-per-pulse measurements [26]. In another study, the SiC Schottky diode was tested as a potential in vivo dosimeter for radiotherapy applications [27]. Diamond-based detection is also used in pulsed dose measurement of linear accelerators in radiotherapy [28].

So far, several studies have been carried out related to the development of both UV and ionizing radiation dosimeters using microfibers or textile materials [29,30,31,32,33,34,35]. The first published results concerned polyamide woven fabric volume-modified with a solution of 2,3,5-triphenyltetrazolium chloride (TTC) [29]. This fabric turned red when exposed to UV light, and after heterogeneous irradiation, the dose distribution could be recorded; for the first time, an attempt was made to measure the 2D UV dose distribution using a textile dosimeter based on TTC-modified polyamide. As a result, further research was carried out; the conclusions are as follows: (i) TTC-containing polyamide fabric can be additionally modified with polymer layers (e.g., latex) to increase resistance to, among others, humidity, (ii) NBT can also be used for such modification of polyamide textile [30]; however, the dose sensitivity to UV light of TTC-polyamide is higher than that of NBT-polyamide, while the dynamic dose response range is higher for NBT-polyamide; therefore, a greater range of dose distribution can be measured with NBT-polyamide than TTC-polyamide, (iii) it is possible to measure the dose distribution after heterogeneous irradiation using ionizing radiation; the first such attempt was carried out with TTC-polyamide exposed to a ^192^Ir high dose rate brachytherapy source [31], (iv) the fabric structure and scanning parameters using a flatbed scanner influence the image noise, which propagates on the dose distribution measurements [32,33], (v) in addition to the volume modification of polyamide textiles, it is possible to produce radiation-sensitive microfibers; polyacrylonitrile microfibers doped with compounds sensitive to UV and ionizing radiation were developed, which were also used to manufacture flat textiles sensitive to radiation [34,35], (vi) screen printing may be used to reduce the consumption of textile modifying solution containing a radiation-sensitive compound; this approach has been successfully tested on cotton and polyamide fabrics using radiochromic physical hydrogels and nitrotetrazolium blue chloride as a UV-sensitive compound, and (vii) screen printing can also be used to modify the surface of cotton fabric to obtain a multi-colored decorative patterns created under the influence of UV light (sensors and dosimeters). However, in all these studies, the cotton fabric was not volumetrically modified and was not evaluated as a 2D dosimeter. Moreover, at the beginning of the research described above, no dedicated software package existed for the rapid processing of such 2D textile dosimetry data. These issues have been resolved, and the results are reported in the current study.

**Figure 1:**
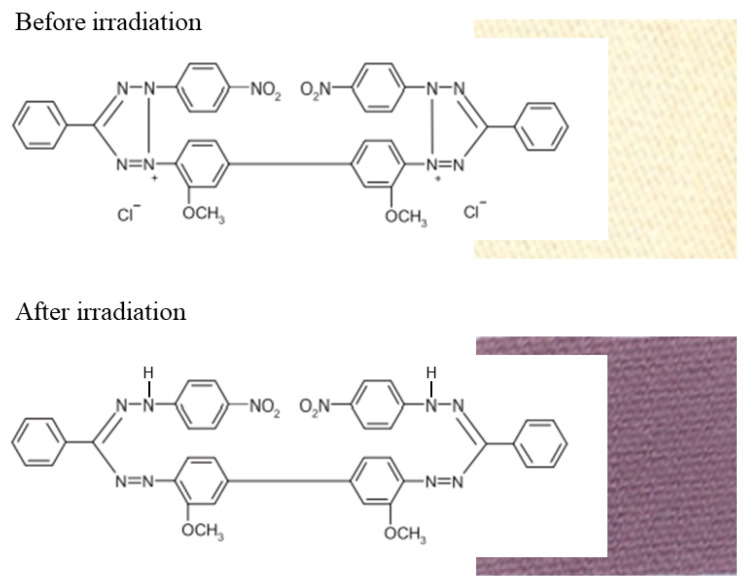
Scheme of reaction of modified cotton fabric under the influence of ionizing radiation. Yellow nitrotetrazolium blue chloride (NBT) in cotton textile transforms into purple-brown color formazan.

The aim of this work was to develop a flexible 2D dosimeter based on textile material through simple chemical modification using a chemical solution with a reduced composition to make it ecological (an aqueous solution of NBT). Therefore, a textile material made of widespread cotton was used, which guaranteed flexibility, and its modification was carried out using an aqueous solution of only one radiation-sensitive compound. The chosen volumetric modification method is padding-squeezing-drying, which allows to produce a textile dosimeter on a large scale, even kilometers in size. The basis of the radiation reaction is the conversion of a radiation-sensitive compound, nitrotetrazolium blue chloride, into a colored formazan (Figure 1). The following are investigated: (i) the dose response of the dosimeter in the kilogray range, (ii) the effect of dose rate on color development, (iii) the effect of data processing on the dose resolution of the dosimeter, (iv) heterogeneous irradiation of the dosimeter to assess the possibility of 2D dose resolution measurements, (v) the possibility of using such a dosimeter to measure dose distribution on non-flat surfaces and, in general, (vi) testing a system consisting of a textile dosimeter combined with flatbed scanner reading and dedicated software packages as a comprehensive dosimetric tool. Due to the large dose range and high dose rates to which the dosimeter responds, it is intended for use in industrial processes using ionizing radiation rather than, for example, for radiotherapy purposes, where a much smaller dose range and dose rates are used.

## 2. Results and Discussion

### 2.1. Impact of Dose and Dose Rate

If NBT-modified cotton fabric is irradiated, it turns purple-brown. The intensity of the color depends on the dose absorbed; the higher the dose, the more intense the color. The same color change was observed regardless of the radiation dose rate applied. The color comes from the conversion of NBT to formazan and is less likely to be influenced by the color change of pure cotton when exposed to ionizing radiation. Elsewhere, it was shown that irradiation of cotton with 25 kGy caused only a slight color change, and the color difference was −0.74 (ΔL*) [36].

**Figure 2:**
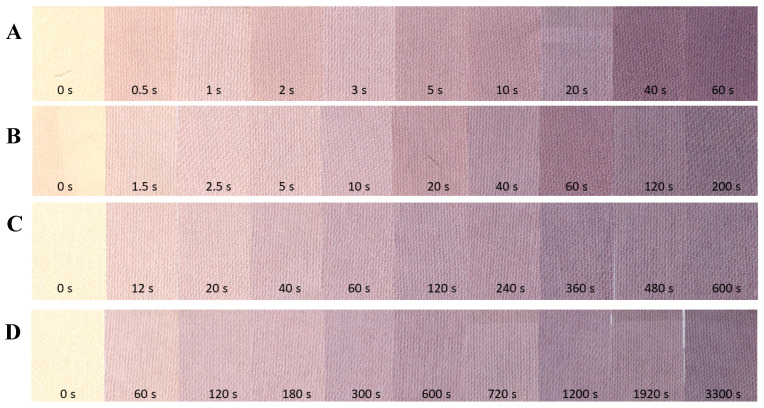
Images of samples scanned (modified cotton—2D dosimeter) with an HP Scanjet flatbed scanner, which were exposed to accelerated electrons. The samples were located at the following distances from the accelerator beam outlet: 50 (**A**), 80 (**B**), 155 (**C**), and 270 cm (**D**) corresponding to a dose rate of 73.1 (**A**), 22.7 (**B**), 4.9 (**C**) and 1.1 kGy/min (**D**). The exposure times are given in the images.

**Figure 3:**
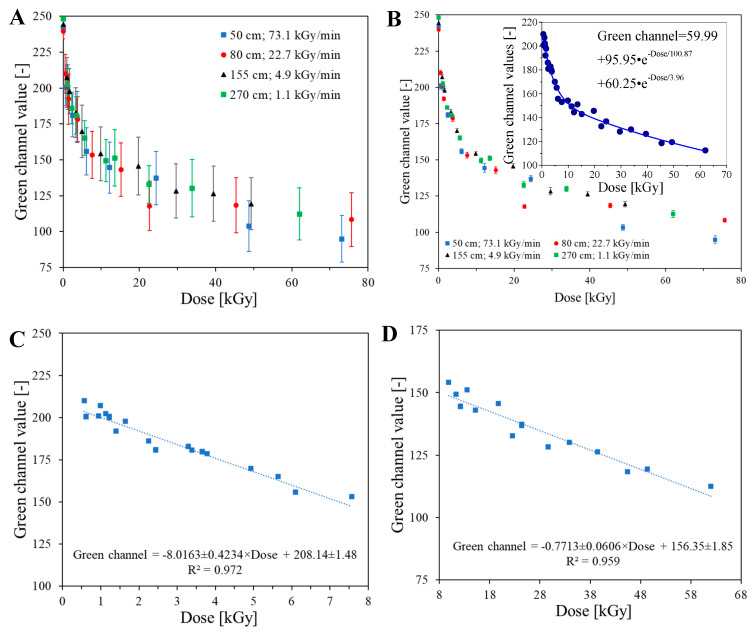
Response of samples to irradiation for samples located at different distances from the accelerator beam outlet and thus exposed to different dose rates. The change in color of the samples is expressed as the values of the green channel of the RGB color model. The green channel values are calculated for a region of interest of 7000–10,000 points. Graph (**A**) is for a sample scanned with an HP Scanjet flatbed scanner without the mean filter applied, graph (**B**) is for the same data after applying the mean filter (kernel size: 3, kernel unit: mm, kernel mode: 2D, iterations: 2), the inset in (**B**) is exponential decay (2nd order) fitting all calibration points, assuming no dose rate dependence for NBT-modified cotton samples. Graph (**C**,**D**) illustrate the effect of fitting linear functions to all points, as in the inset in (**B**), but for different absorbed dose ranges (inset (**B**,**D**) do not include the points at 22.7 and 48.8 kGy, which clearly deviate from the general trend).

Irradiated samples at different dose rates ranging from 1.1 to 73.1 kGy/min were scanned using a flatbed scanner, and the results are shown in Figure 2. As can be inferred from the images, the weave structure of the cotton fabric can be easily seen. Interestingly, this is more clearly visible when scanned than with the naked eye, despite the scanning setting being set to 75 dpi. It was assumed that this feature may be a problem when using a dosimeter for high-resolution 2D dose distribution measurements. This was confirmed after preparing calibration relationships between the values of the green channel of the RGB color model and the absorbed dose, as shown in Figure 3. Each point in Figure 3A is the mean value of the entire sample for the region of interest of 7000–10,000 points, and standard deviation bars are also drawn. Their high values result from the fact that the structure of the cotton fabric weave produces significant noise. To reduce this, the mean filter was applied, and the results obtained are presented in Figure 3B. The standard deviation values have been significantly reduced, and the corresponding bars are often covered by the points. After analyzing the dose response of the 2D cotton dosimeter, the following conclusions can be drawn: (i) the dynamic dose response is up to about 80 kGy, (ii) there is no dependence of the dosimeter on the dose rate in the examined range (Figure 3A,B), and (iii) the decrease in the value of the green channel as a function of the absorbed dose can be described by the function given in the inset of Figure 3B; however, the same relationship can be divided into two linear dose response ranges: 0.6–7.6 kGy and 9.9–62.0 kGy, for which linear calibration equations were obtained (Figure 3C,D); those equations indicate that initially the dosimeter turns purple-brown with a dose sensitivity of −8.0163 ± 0.4234 kGy ^−1^ (R^2^ = 0.972, SD = 4.082, N = 23, *p* < 0.0001), and then the color conversion slows down and the dose sensitivity is much lower: −0.7713 ± 0.0606 kGy^−1^ (R^2^ = 0.959, SD = 3.663, N = 16, *p* < 0.0001). The dose sensitivity is the decrease in the green channel value corresponding to an increase in dose of 1 kGy. The exact cause of the observed significant decrease in dose sensitivity at approximately 8 kGy is unknown. However, there is no doubt that the NBT substrate is depleted during irradiation, and most of the substrate appears to be converted to formazan up to approximately 8 kGy. As a result, the color of the samples does not change so intensely above 8 kGy. One can only speculate that at higher doses, NBT substrate residues are still converted to formazan, but in parallel, NBT formazan may be degraded, which overall results in the observed decrease in dose sensitivity. Tetrazolium salt bleaching has also been observed elsewhere in UV-exposed samples [29]. Nevertheless, this phenomenon requires further research.

**Figure 4:**
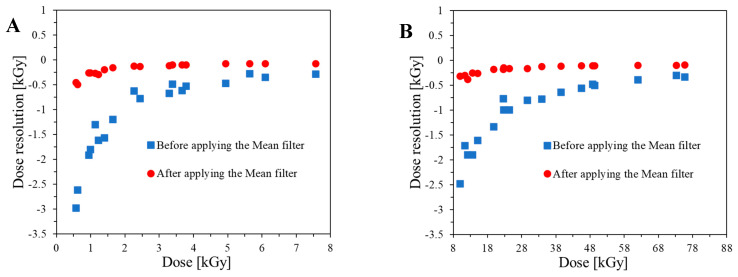
Dose resolution calculated for the 2D dosimeter (NBT-modified cotton) for the lower (**A**) and higher (**B**) dose range; two linear dose ranges are presented in Figure 3C,D. The dose resolution was calculated for samples after scanning with the HP Scanjet flatbed scanner before and after filtration (the mean filter, kernel size: 3, kernel unit: mm, kernel mode: 2D, iterations: 2).

Image processing of scanned modified cotton samples affects the dose resolution of such dosimeters (Figure 4A,B). Dose resolution was calculated according to the approach presented elsewhere [37,38]. For unfiltered images (based on calibration data in Figure 3A), the dose resolution is much lower than for filtered images (based on calibration data presented in Figure 3B). In consequence, when modified cotton is used for 2D dose distribution measurements, filtering of the images for the dosimeter after exposure must be performed, which significantly improves the dose resolution of the dosimeter.

Modified cotton samples are relatively stable during storage. However, a slowly occurring color change was observed. Within 11 days and 14 h, the color of the samples increased by 1.4% from the initial value, which corresponds to approximately 0.005%/h of storage. For this reason, a dosimeter can be used within a few months of manufacture, but determining accurate validity data requires long-term experiments (months to years), which was not assessed in this study.

### 2.2. 2D Dose Distribution

**Figure 5:**
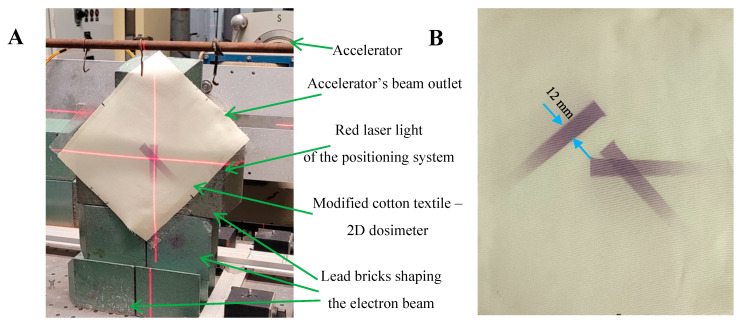
Photographs illustrating the irradiation setup of the modified cotton sample (**A**) and the resulting arbitrary chosen pattern of dose distribution obtained for the modified cotton sample (**B**) (sample dimensions: 22 × 18 cm^2^).

**Figure 6:**
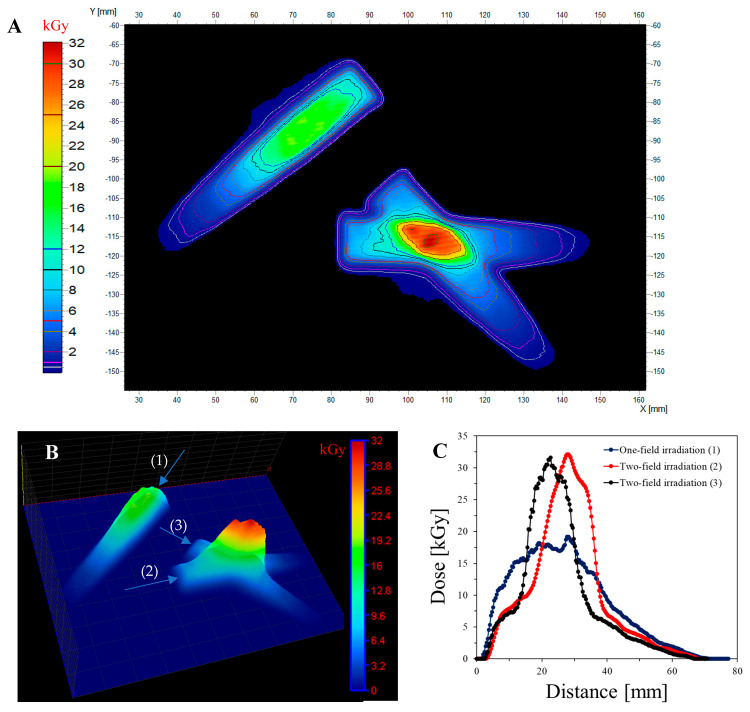
2D dose distribution measured with a flexible 2D dosimeter (NBT-modified cotton) located in front of the electron beam at a 50 cm distance from the beam outlet, as shown in Figure 5A. (**A**): 2D dose distribution map with isodoses superimposed; the irradiation pattern corresponds to that in Figure 5B. (**B**): 3D illustration of a 2D dose distribution map; (1), (2), and (3) indicate the starting positions from which the dose profiles were prepared and are illustrated in (**C**). Data processed in the polyGeVero (v. 2.0) and polyGeVero-CT (v.1.2) software packages.

**Figure 7:**
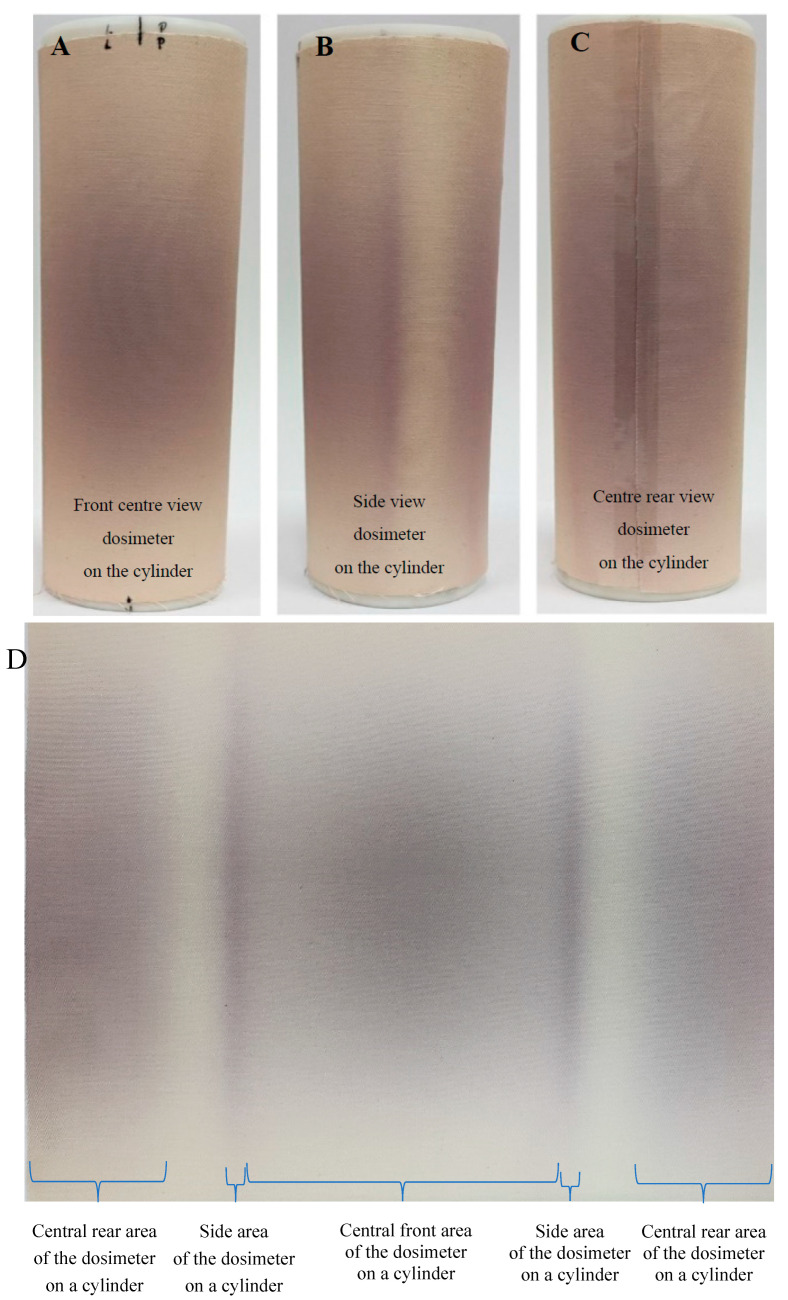
Photographs of a modified cotton sample wound on a plastic cylinder (2D dosimeter on a cylinder); (**A**): view from the accelerator beam exit side, (**B**): side view, at an angle of 90° to the beam outlet from the accelerator, (**C**): view of the rear part of the cylinder, at an angle of 180° to the accelerator beam (note that a stripe of adhesive tape is seen centrally along the cylinder, which fixed the sample on the cylinder). (**D**): View of the sample unwound from the cylinder; the central part of the sample is the part directed toward the outlet of the accelerator beam (dimensions of the sample 26.5 × 22 cm^2^).

**Figure 8:**
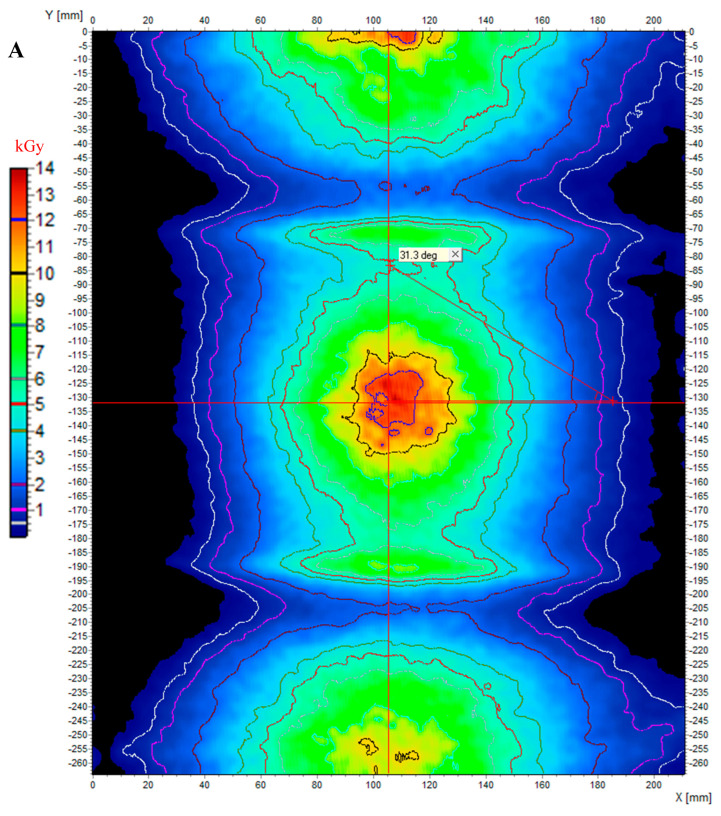

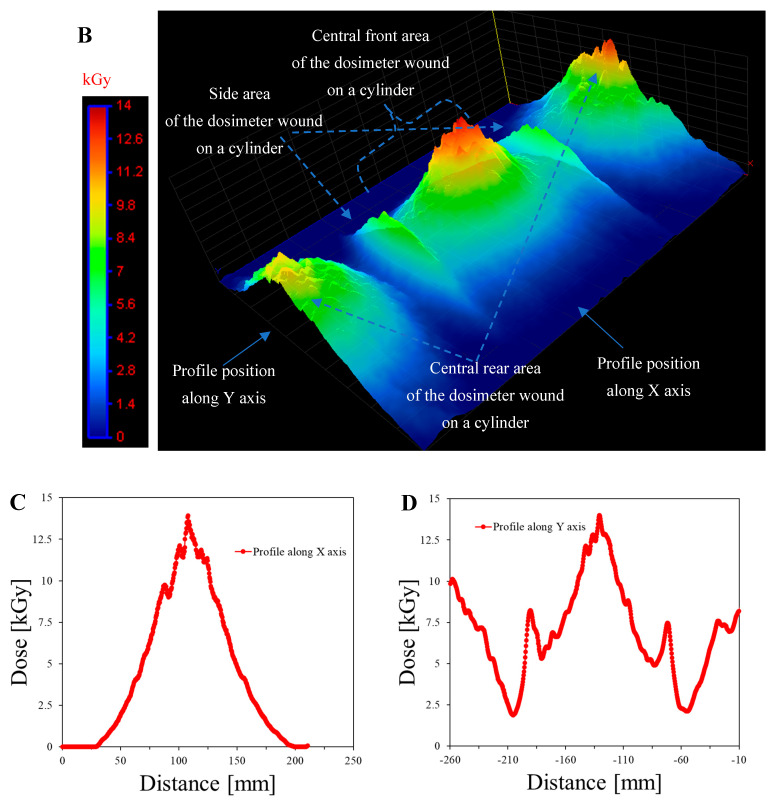
2D dose distribution measured with a flexible 2D dosimeter (NBT-modified cotton) located in front of the electron beam at a 50 cm distance from the beam outlet; dosimeter wound on a plastic cylinder as shown in Figure 7A–C. (**A**): 2D map of dose distribution with isodoses superimposed (image is a 90° clockwise rotation of the image shown in Figure 7D); two red lines along the X and Y axes intersecting in the center of the image locate the center of the beam; a protractor is drawn that indicates 31.3° from the beam axis, which is the extent of the irradiation effect at the front of the sample. (**B**): 3D illustration of a 2D dose distribution map with indications of the profiles along the X and Y axes; profiles are presented in (**C**,**D**). Data processed in the polyGeVero (v. 2.0) and polyGeVero-CT (v. 1.2) software packages.

The modified cotton samples were irradiated in a heterogeneous manner. Two approaches were implemented for this purpose. For the first one, in which the sample was placed in front of the outlet of the accelerator beam, the beam was shaped (limited) using lead bricks (Figure 5 and Figure 6). For the second one, the sample was wound on a cylinder and irradiated without shaping the electron beam (Figure 7 and Figure 8). In the first case, by turning the sample after each exposure, three colored stripes were obtained. The configuration and effect of the irradiation are visible in Figure 5A,B. In Figure 5B, the sharp shapes of the three colored stripes, one separate and two crossed, are visible. The shapes of the stripes are durable and do not blur or fade within a month of storage. In general, irradiated samples are relatively stable during storage. It has been calculated that during 11 days and 14 h of storage, the color increases by approximately 6%, which corresponds to approximately 0.02%/h of storage. For this reason, scanning can be performed within hours after irradiation, or even weeks or more than a month, without affecting the reading of samples.

An irradiated three-stripe cotton sample was scanned using a flatbed scanner, the obtained image was separated in RGB channels, and the green channel values were converted into dose values after applying the calibration relationship given in the inset of Figure 3B. The result of this operation in the form of 2D dose distribution maps is presented in Figure 6A,B. In Figure 6A, the isodose lines are also superimposed on the dose distribution map. In Figure 6B, the dose distribution map is presented using a 3D plane dose distribution option, additionally enabled by the polyGeVero software package. In Figure 6C, three profiles are presented along the irradiated regions, as indicated in Figure 6B. The profile for one-stripe irradiation indicates a maximum dose of approximately 17 kGy. Profiles for two-stripe irradiation indicate a maximum dose that is approximately twice the dose for one-stripe irradiation. This is consistent with the exposure time being similar for irradiating each stripe. This experiment conclusively proved that the modified cotton could be used as a 2D radiation dosimeter to measure shaped radiation beams.

The second approach was to demonstrate the potential of modified cotton as a flexible 2D dosimeter. A large sample with dimensions of 26.5 × 22 cm^2^, wound on a cylinder and unwound, is presented after irradiation in Figure 7. The electron beam passing through the sample caused a color change, with the color being the most intense in the center of the sample (beam axis), at the front and back, and decreasing in color intensity at the top and bottom of the sample (Figure 7A,C). An interesting color development was observed on both the left and right sides of the sample, as seen in Figure 7B,D. An increase in the absorbed dose can be observed on both sides of the cylinder (at an angle of approximately 31° to the incident beam, as shown with the protractor in Figure 8A) and at further distances to the back of the sample, the absorbed dose decreases. We can speculate about the increase in color intensity on both sides, which may be related to the depth-dose effects of electron absorption and the several millimeters thickness of the cotton fabric in these places. Because the cotton fabric was wound on a plastic rather than a metal cylinder, the possibility of bremsstrahlung or X-rays formation, which could increase the dose, and thus the intensity of the color, was ruled out. In turn, the reduction in absorbed dose at further distances to the back of the sample is the result of the absorption of the electron beam by the front and rear walls of the plastic cylinder material.

After irradiation, the sample was scanned using a flatbed scanner, the resulting image was filtered (the mean filter applied) and converted to dose distribution, as previously. The results of these operations are presented in Figure 8. In Figure 8A, a 2D dose distribution map is visible with isodose lines superimposed. The obtained dose distribution thoroughly reflects the color changes described above. In Figure 8B, the dose distribution map is presented using a 3D plane dose distribution option, analogous to the results for the irradiated sample with three stripes. In Figure 8C,D, exemplary profiles are presented (in arbitrary chosen positions) along and across the middle part of the sample. The profiles indicate areas of increased dose at the front of the sample and decreased dose at the rear of the sample relative to the front, as well as reduced dose responses on the sides of the sample due to the above-mentioned absorption of electrons by the plastic cylinder material.

After both experiments, the following conclusions can be drawn: (i) the modified cotton fabric suits perfectly in cases where measurements of 2D dose distribution on the surfaces of various objects, including cylindrical ones, are required, (ii) the adopted data processing method, which includes image filtering, allows for the reduction in image noise and obtaining high-resolution dose distribution maps, and (iii) the developed dosimeter, combined with a scanning method and data processing by the polyGeVero-CT and polyGeVero software packages, constitutes a comprehensive 2D dosimetric analysis tool.

## 3. Materials and Methods

### 3.1. Preparation of Samples

**Figure 9:**
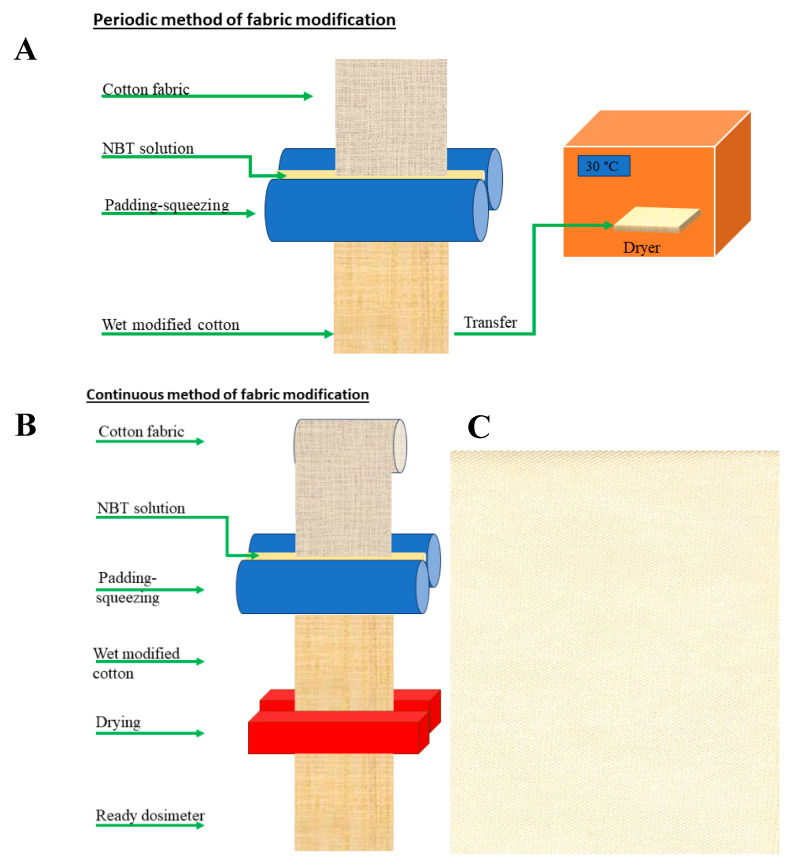
Scheme illustrating the manufacturing possibilities of a 2D dosimeter from cotton textile (**A**,**B**) and a scan of the dosimeter after preparation (ready for irradiation); the structure of twill weave can be discerned (**C**); scanning was performed with an HP Scanjet G3010 scanner.

Cotton textile samples (white woven fabric, not brightened, warp: 240/dm, weft: 220/dm, twill weave, surface weight: 250 g/m^2^, and thickness of 0.68 mm, Royal TenCate, Nijverdal, The Neverlands) were pre-prepared by washing in 1 g/dm^3^ of non-ionic surface-active agent (Rokafenol N8-P7, Boruta Zgierz, Zgierz, Poland) for 30 min at 40 °C. This was to remove impurities deposited during the storage of a roll of cotton fabric. Afterwards, they were rinsed with tap water and distilled water and dried at 40 °C for 120 min. After ironing, they were ready for chemical modification. There are two possibilities for the production of volume-modified cotton: periodic and continuous (Figure 9A,B). In the case of the periodic method (Figure 9A), smaller sample sizes can be made in two steps, including padding-squeezing (1st stage), and drying (2nd stage). A transfer of wet modified cotton from the first stage to the second stage is required. For continuous manufacturing, transfer is not required. Long cotton fabric can be produced. The cotton fabric is introduced into the padding-squeezing by unwinding the fabric from the beam, and after the first stage is completed, another drying stage occurs continuously (Figure 9B). In the current work, we chose the periodic modification method because there was no need for large quantities of modified cotton production. Therefore, the padding-squeezing machine was used of E. Benz (Stuttgart, Germany). Cotton samples were soaked in a 10% nitrotetrazolium blue chloride solution (NBT, M = 817.64 g/mol, Roth, Karlsruhe, Germany) and squeezed (clamp 45 kG/cm, rotation speed 4 m/min). Afterwards, they were dried at 30 °C for 120 min (Figure 9). The weight of the cotton sample (10.0 ± 0.1 cm^2^) before chemical modification, after soaking, squeezing and drying was equal to 2.3250, 5.4831, 3.4421, and 2.4832 g, respectively. The cotton samples prepared in this way were wrapped in aluminum foil to protect against daylight and stored at room temperature until irradiation.

### 3.2. Irradiation

**Figure 10:**
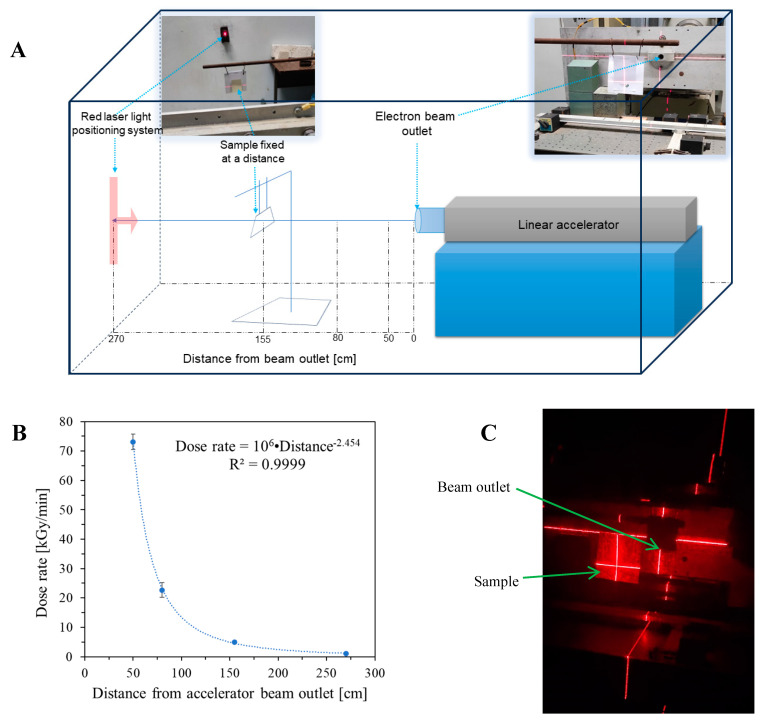
Experimental setup for irradiation of modified cotton samples (**A**) and experimental results of dose rate measurements at the beam axis at specific distances (marked in (**A**)) from the accelerator beam outlet (**B**). Subfigure (**C**) illustrates the positioning of modified cotton samples using a red-light laser positioning system.

A linear accelerator (ELU 6-E Elektronika, Moscow, USSR) was used for homogeneous and heterogeneous irradiation of modified cotton samples. Irradiation occurred approximately 21–27 h after sample preparation. The samples were irradiated in air and positioned perpendicular to the electron beam (6 MeV) (Figure 10A). A semicontinuous irradiation with pulses of 4 µs duration generated with 20 Hz was applied. Initially, three dosimetric systems were employed to measure the dose, graphite calorimetric dosimeters (HRRL, Lyngby, Denmark, RISO calibrated), alanine pellet dosimeters (EPR e-scan Bruker, Billerica, MA, USA, according to ISO/ASTM 51607:2013) and film dosimeters (B3 WINdose Dosimeters, average thickness: 0.0190 ± 0.0003 mm, GEX Corporation, Palm City, FL, USA). The readings from these dosimeters after 10 min of exposure to an electron beam at 270 cm distance from the accelerator outlet were 11.8, 10.9, and 11.1 kGy for calorimetric, alanine and film dosimeters, respectively. As the calorimetric and (to lower extent) pellet alanine dosimeters are essentially dedicated for volumetric dose determination, the film dosimeter was used to monitor the delivered dose in all further experiments, because it measures the surface dose, similar to the examined thin cotton textile dosimeter. One should note that the maximum energy deposition of 6 MeV electrons should be at ca. 10 mm in 1 g/cm^3^ density material. The 2D cotton dosimeters samples were exposed to irradiation with four different dose rates regulated by the distance from the electron beam outlet (50, 80, 155, and 270 cm), as illustrated in Figure 10A. The doses read from the film dosimeters were divided by the irradiation times at specific distances from the beam outlet. Thus, dose rates of 1.1 ± 0.1, 4.9 ± 0.5, 22.7 ± 2.5, and 73.1 ± 2.6 kGy/min were obtained for 270, 155, 80, and 50 cm, respectively, from the beam outlet. The dose rate decreases with the distance from the beam outlet (due to intrinsic repulsion of electrons bearing elemental charge), which can be described by the power function and is presented in Figure 10B.

The modified cotton samples were subjected to uniform irradiation at any distance from the beam outlet (four dose rates) in order to obtain calibration relationships: color change versus the absorbed dose. The irradiation of each modified cotton sample was related to the irradiation of the film dosimeter that was attached to the back of the cotton sample. Both the cotton sample and the film dosimeter were aligned relative to the center of the electron beam outlet using a red-light laser positioning system (Figure 10C). The dose read from the film dosimeter was correlated with the color change of this dosimeter to obtain a calibration relationship. In the case of non-uniform irradiation of the modified cotton, the samples were irradiated at a distance of 50 cm from the beam outlet. Two irradiation approaches were tested. The first was to irradiate a flat sample so that it was perpendicular to the electron beam. Lead bricks were placed to create a gap and narrow the electron beam; a gap of roughly 9.0 × 1.2 cm^2^ was created. The sample was irradiated three times: the first time it was exposed only once to create one colored stripe, and the second time it was irradiated twice to create two intersecting, colored stripes (after the first exposure the sample was rotated). The second irradiation approach involved preparing a sample of modified cotton wound on a plastic cylinder (height: ~23 cm, diameter: 8.3 cm, thickness: 0.4 cm). Afterwards, the cylinder with the sample was placed at a distance of 50 cm from the outlet of the electron beam and irradiated. The distance of the cylinder from the beam outlet and the size of the sample on the cylinder guaranteed non-uniform irradiation of the sample. After irradiation, the cotton dosimeter was unwrapped, and both heterogeneously irradiated samples were scanned as described in Section 3.3.

### 3.3. 2D Measurements

All dosimeter samples were scanned using a flatbed scanner (HP Scanjet G3010). The settings were as follows: 75 dpi, brighten/darken option: 11 (brighten), −69 (shadows), 0 (intermediate shades), image sharpening: none, color adjustment: none (color saturation 100%), automatic color correction: none. The generated dosimeter sample bitmaps were further processed as described in Section 3.4.

### 3.4. Data Processing

**Figure 11:**
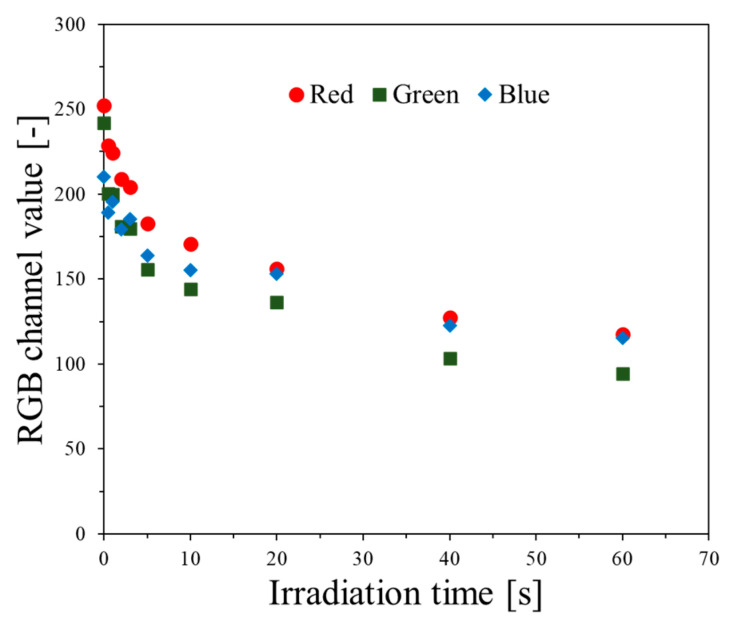
Contribution of RGB color model channels to the color changes of modified cotton samples after irradiation (73.1 kGy/min).

After scanning the dosimeters with the HP Scanjet G3010 scanner, the data were processed using the polyGeVero^®^-CT (v.1.2, GeVero Co., Lodz, Poland) software package. The polyGeVero^®^-CT software was initially designed to process 3D radiotherapy dosimetry with computed tomography readings [39]. However, recently, it has been enriched with functionalities related to 2D image processing. The images were decomposed to observe the red, green, and blue channels in the RGB color model, depending on the irradiation time (Figure 11). All channels contribute substantially to the color changes of irradiated samples. The color difference between non-irradiated and irradiated samples for 60 s (73.1 kGy/min) was 53.3, 60.9, and 45.1% for the red, green, and blue channels, respectively. On this basis, the green channel was selected as the one that most influenced the color change of the samples after irradiation. Using the tools of the polyGeVero-CT software package, the images were also filtered; the mean filter was applied at various settings. In conclusion, the following settings were used: kernel size: 3, kernel unit: mm, kernel mode: 2D, iterations: 2. They allowed the image to be smoothed without losing its shape. The smoothed images were transformed into 2D dose distribution images after applying the calibration equation (obtained using the least-squares algorithm [40]). These 2D dose maps were exported to the polyGeVero^®^ software package (v. 2.0, GeVero Co., Lodz, Poland) [41] for further analysis.

## 4. Conclusions

This work concerned a flexible 2D dosimeter. It is designed to measure a wide range of radiation doses (kilograys) during exposure to radiation beams with a wide range of dose rates. The dosimeter can be easily produced by volume modification of cotton fabric using the padding-squeezing-drying method. For this purpose, an aqueous solution of nitrotetrazolium blue chloride is used as a radiation-sensitive compound. No other complex chemical system was used; chemical modification was deliberately reduced to only the necessary compound. Therefore, the manufacturing method itself and the obtained dosimeter can be regarded as ecological.

It has been shown that the dosimeter responds to non-uniform irradiation, and the color distribution is stable during storage. A 2D scanning method with a flatbed scanner was proposed, and the data were processed using dedicated software packages allowing for quick conversion of 2D images into 2D dose distribution after using the calibration function. It is expected that the dosimetric system may be used to measure 2D dose distribution in industrial processes using ionizing radiation and to monitor dose distribution for packaging of various shapes. Due to the large dose range and high dose rates to which the dosimeter responds, it is not intended for, for instance, radiotherapy purposes, where a much smaller dose range and dose rates are employed. It should be noted that such a dosimeter can be mass-produced with any dimensions and even kilometers in length (as illustrated in Section 3.1), using typical wet processing and chemical finishing machines for continuous processes in the textile industry.

## Data Availability

The data supporting the reported results are not stored in any publicly archived datasets. The readers can contact the corresponding author for any further clarification of the results obtained.

## Abbreviations

2D	two dimensional
3D	three dimensional
CT	computed tomography
dpi	dots per inch
HP	Hewlett-Packard
NBT	nitrotetrazolium blue chloride
RGB	Red-green-blue
TTC	2,3,5-triphenyltetrazolium chloride
UV	ultraviolet radiation
